# Supplementary figures and images for: KRAS inhibition reverses chemotherapy resistance promoted by therapy-induced senescence-like in pancreatic ductal adenocarcinoma
Source: Transl Oncol. 2025 May 17;57:102421. doi: 10.1016/j.tranon.2025.102421 (PMC12143771; doi:10.1016/j.tranon.2025.102421)

## Slide 1
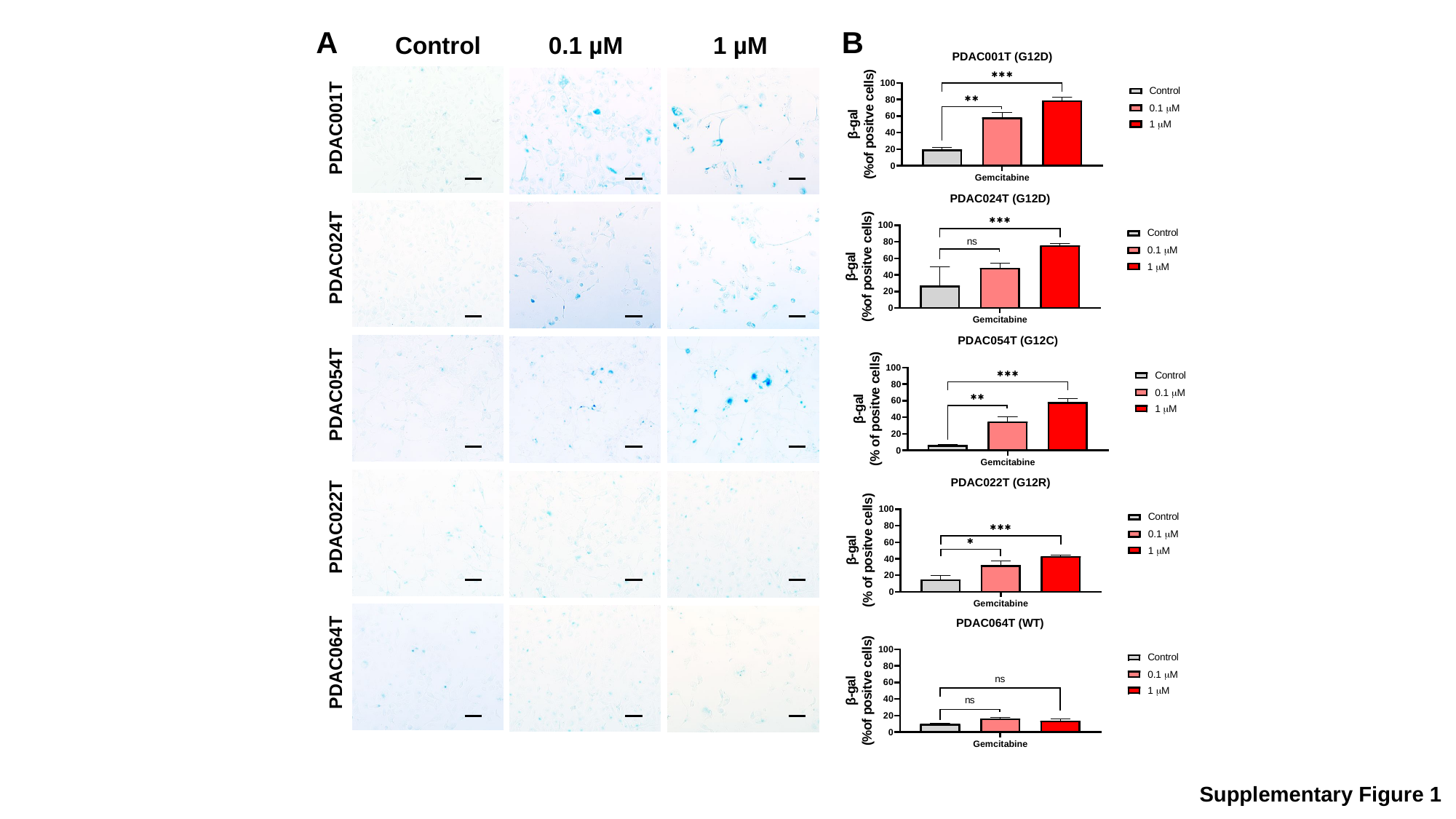

A
B
Control
0.1 µM
1 µM
PDAC001T
PDAC024T
PDAC054T
PDAC022T
PDAC064T
Supplementary Figure 1

Supplement: Supplementary file 1 [file mmc1.pptx]

## Slide 1
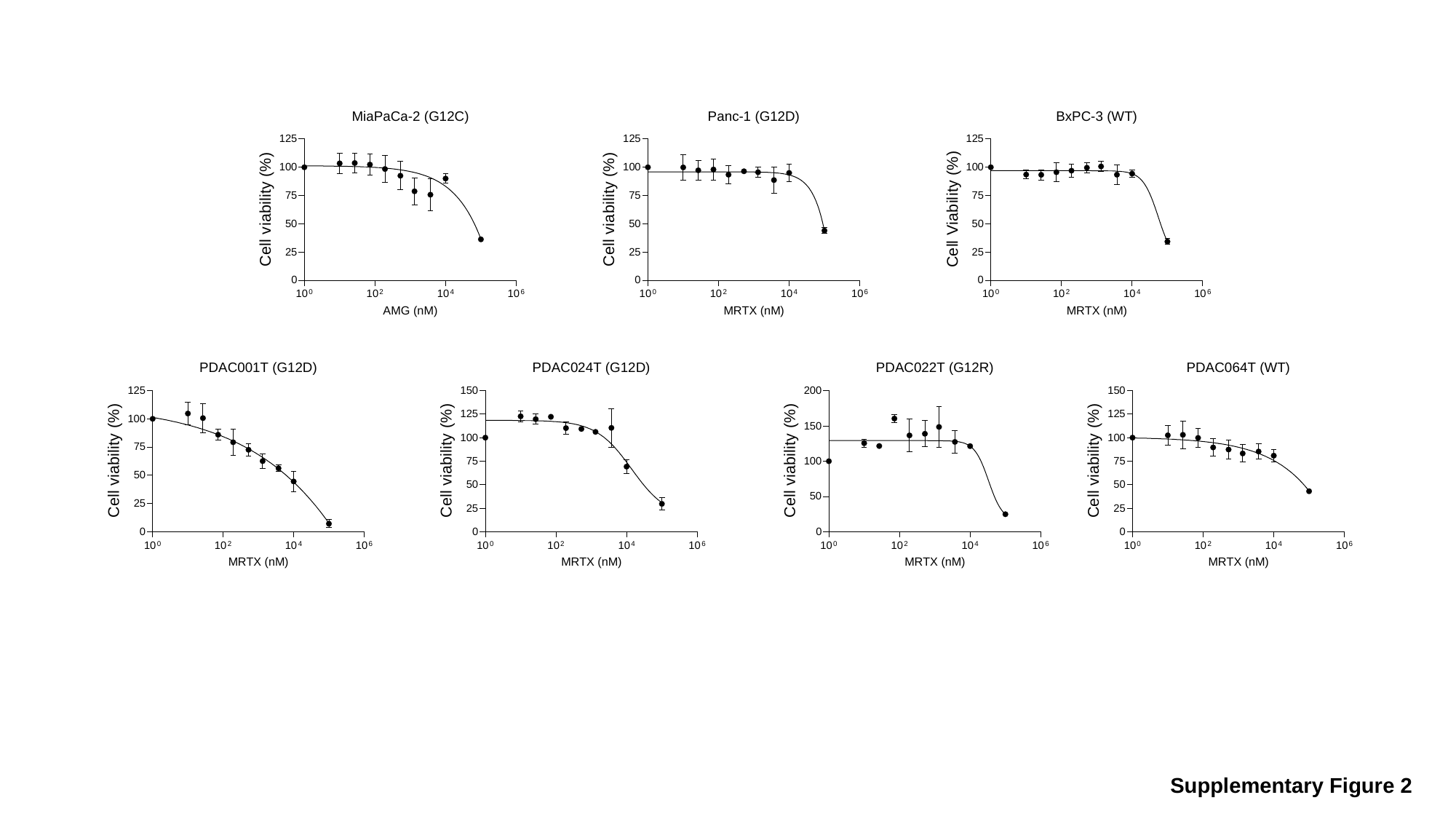

Supplementary Figure 2

Supplement: Supplementary file 2 [file mmc2.pptx]
